# An Innovative Mathematical Model: A Key to the Riddle of HbA_1c_

**DOI:** 10.1155/2010/481326

**Published:** 2010-08-29

**Authors:** Mahdi Kahrom

**Affiliations:** Division of Endocrinology and Metabolism, Bu-Ali Research Institute, Mashhad University of Medical Sciences, Mashhad 9195977178, Iran

## Abstract

HbA_1c_ is a standard clinical assessment of glycemia and the basis of most data relating glycemic control to complications. While daily blood glucose testing gives a picture of day-to-day fluctuations, the HbA_1c_ test offers an overview of how well glucose has been controlled over the past 4 months. I devised an innovative mathematical model to describe novel equations governing HbA_1c_ which enables analysis of HbA_1c_ behavior and provides emerging new concepts in assessment of diabetes management. Linear relationship of HbA_1c_ and mean plasma glucose along with the kinetic analysis of HbA_1c_ formation has been used as the basic suppositions to construct this model. The main application of this devised model is prediction of mean plasma glucose at any desired point in time after a change in therapy and with great certainty. This model also appraises the pattern of HbA_1c_ changes over time and provides a unique opportunity to address common mistakes and misconceptions in routine application of HbA_1c_ that could have potentially important implications on diabetes control.

## 1. Introduction

Maintenance of blood glucose levels as close as possible to the nondiabetic range over time is an important goal in the current management of patients with diabetes. Assessment of a patient's diabetes management can be accomplished by directly analyzing the pattern of multiple blood glucose samples drawn over time [1]. However, a high degree of cooperation is required on the part of the patient to collect a sufficient number of blood glucose samples that adequately represent typical diurnal glucose patterns. Once collected, statistical analysis is then necessary to assess the central tendency and variability of glucose levels. As an alternative, a patient's HbA_1c_ level can be easily and conveniently determined from a single blood sample. 

A large number of studies have shown that HbA_1c_ is strongly associated with the preceding mean plasma glucose (MPG) over previous weeks and months [2–5]. Based on the statistical relation of HbA_1c_ and MPG, HbA_1c_ is widely used as a clinical estimation of MPG, and it has been proposed as a diagnostic criterion for diabetes, as well [6]. HbA_1c_ has, therefore, become a standard assessment of glycemia [7] and a standard part of diabetes management.

One of the most important limitations of HbA_1c_ is that it is not applicable in short intervals. Erythrocyte life span in normal conditions averages ~120 days, and the glycation of hemoglobin (Hb) is a continuous, nonenzymatic, relatively slow and nearly irreversible process [8] that means change in effects of previous glycation on Hb takes several weeks to months to occur. To permit a much clearer assessing of diabetes management, it is generally recommended that the HbA_1c_ assay be used every 2-3 months. Ideally, if measured each 120 days (4 months) it gives a precise estimation of MPG over preceding 4 months, reliably comparable to previous HbA_1c_ value. If measurement is taken earlier than erythrocyte life span intervals (4 months), because of existing previously glycated Hbs which have not reached end of their lives, the estimated MPG would be affected by previous plasma glucose levels. This would be an important issue, and if measured following a significant variations in plasma glucose during changes in patient's diabetes control or medication, then it would end to a remarkable error in estimation of MPG.

I devised an innovative mathematical model to describe novel equations governing HbA_1c_ which enables analysis of HbA_1c_ behavior and provides emerging new concepts in assessment of diabetes management.

## 2. Suppositions and Theory

**Figure 1:**
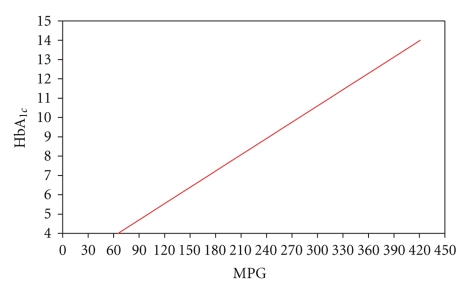
Linear regression analysis of MPG versus HbA_1c_: the Pearson correlation coefficient (*r*) is 0.82; MPG  (mg/dl) = 35.6 × HbA_1c_ − 77.3.

Quarterly HbA_1c_ and corresponding seven-point capillary blood glucose profiles obtained in the DCCT have been analyzed to define the relationship between HbA_1c_ and MPG. HbA_1c_ is linearly related to MPG based on linear regression analysis weighted by the number of observations per subject (Figure 1), producing a relationship of [9]

(1) $$\begin{array}{c} \text{MPG}\left(\text{mg}/\text{dl}\right)=35.6\times {\text{HbA}}_{1\text{c}}-77.3. \end{array}$$

or

(2) $$\begin{array}{c} {\text{HbA}}_{1\text{c}}=\frac{1}{35.6}\text{MPG}+2.17. \end{array}$$

MPG at increasing levels of HbA_1c_ is shown in Table 1 based on DCCT data correlating HbA_1c_ with MPG using 7-point blood glucose profiles along with ADAG data using continuous glucose monitoring systems [5, 9]. 

**Figure 2:**
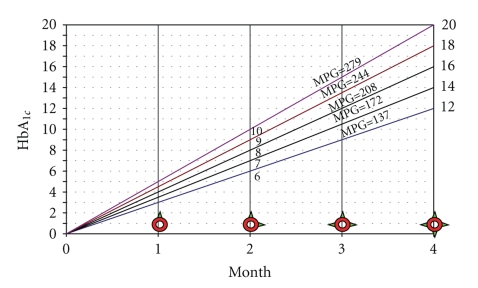
Rate of formation of HbA_1c_ simulated from results of prolonged incubation of HbA_0_ with glucose in different concentration.

The kinetic analysis of HbA_1c_ formation depicted in Figure 2 shows the linear relationship between HbA_1c_ formation rate and time, with the slope proportional to the MPG [10]. The higher the blood glucose is, the faster HbA_1c_ will be formed, resulting in higher HbA_1c_ levels. It also demonstrates the distribution of HbA_1c_ amount in erythrocytes with different ages. For instance, it is elicited from the curve MPG = 137, that the HbA_1c_ rate in newly born RBCs is 0%, and in RBCs with 60 and 120 days old, 6 and 12%, respectively. 

It can be corroborated that the mean HbA_1c_ in a collection of erythrocytes with different ages and HbA_1c_ rates is the median point or arithmetic mean of the upper and lower limits of the curve. Erythrocyte life span in normal conditions is about 120 days and the level of HbA_1c_ at any point in time is contributed to by all circulating erythrocytes, from the oldest (120 days old) to the youngest. Since the rate of RBC formation is equal to its degradation, the percentage of RBC count in a single day is 1/120 of total RBC mass. Hence, 1/120 of RBC collection are one day old, 1/120 are two days old, and likewise 1/120 are 120 days old. The mean value of HbA_1c_ in a collection of RBCs with different ages can be calculated by averaging of HbA_1c_ rate in each RBC as follows:

(3) $$\begin{array}{c} \text{Mean}\;\text{Hb}{\text{A}}_{1\text{c}}=\frac{\sum_{1}^{120}\varepsilon_{n}\times \left(1/120\right){\text{RBC}}_{\text{mass}}}{{\text{RBC}}_{\text{mass}}}, \end{array}$$

where *ε*_*n*_ is HbA_1c_ rate in RBCs with n days old and RBC mass is total number of RBCs in the body. Since glycation of Hb according to Figure 2 follows a linear pattern, it is expected that

(4) $$\begin{array}{c} \varepsilon_{2}=2\varepsilon_{1},\;\;\varepsilon_{3}=3\varepsilon_{1},\ldots ,\;\;\varepsilon_{120}=120\varepsilon_{1}. \end{array}$$

Hence,

(5) $$\begin{array}{cc} {\text{HbA}}_{1c} & =\frac{\left(1/120\right){\text{RBC}}_{\text{mass}}\times \left(\varepsilon_{1}+\varepsilon_{120}\right)\times \left(120/2\right)}{{\text{RBC}}_{\text{mass}}}\\ & =\frac{\left(\varepsilon_{1}+\varepsilon_{120}\right)}{2}. \end{array}$$

Accordingly, the mean value of HbA_1c_ in a collection of RBCs would be the arithmetic mean of upper and lower limits of the curve.

The mathematical relationship between data leading to the curves depicted in Figure 2 can be correlated by the following formula:

(6) $$\begin{array}{c} {\text{HbA}}_{1\text{c}}=\frac{\text{MPG}/35.6+2.17}{2}m, \end{array}$$

where MPG contributes the mean plasma glucose in which Hb glycation is progressing and m is the variation of time in month.

We will now map this model into mathematical expressions and start with an example.

**Example 1**

**Figure 3:**
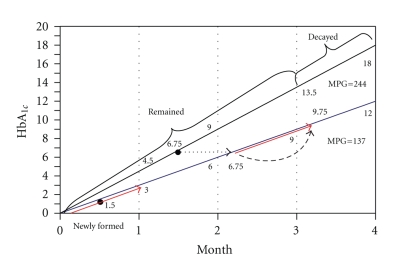
Plotted graph demonstrating process of events happening in the hypothetical Example 1.

Assume that you have visited a diabetic patient with HbA_1c_ = 9% and MPG = 244 mg/dl and after adjusting the medications, patient's MPG has fallen to the curve MPG = 137 as visualized by graphic presentation in Figure 3. After one month you are interested in calculating the HbA_1c_ which is the mixture of previously and newly glycated Hbs and is estimated to be in range of 6% to 9%.

As described in Figure 3, after passing one month of changes in MPG, the erythrocytes with 3 to 4 months old will reach the end of their lives and destroy themselves. The remaining RBCs have HbA_1c_ ranging from 0 to 13.5%. This upper extreme can be calculated as

(7) $$\begin{array}{cc} \Phi_{\text{Upper}} & =\frac{{\text{MPG}}_{1}/35.6+2.17}{2}\left(4-m\right)=\frac{{\text{Hb}}_{1}}{2}\left(4-m\right)\\ & =13.5\%. \end{array}$$

As described before (by (5)) the mean value of HbA_1c_ in this group of RBCs is arithmetic mean of upper and lower extremes of the curve, that is

(8) $$\begin{array}{cc} \Phi_{\text{mean}} & =\frac{\Phi_{\text{Upper}}}{2}=\frac{{\text{MPG}}_{1}/35.6+2.17}{4}\left(4-m\right)\\ & =\frac{{\text{Hb}}_{1}}{4}\left(4-m\right)=6.75\text{\%}. \end{array}$$

Over the past one month, these previously glycated RBCs undergo new glycation on the curve MPG = 137 to convey the prior mean HbA_1c_ to a newly higher point. This displacement of mean HbA_1c_ point on the second curve over *m* months can be written as

(9) $$\Delta_{\Phi_{\text{mean}}}=\frac{{\text{MPG}}_{x}/35.6+2.17}{2}m=\frac{{\text{Hb}}_{x}}{2}m=3\%.$$

The sum of contributions (8) and (9) represents the cumulative mean HbA_1c_ in this group of RBCs with former and later glycation on two different curves.

(10) $$\begin{array}{cc} \Phi_{1} & =\frac{{\text{MPG}}_{1}/35.6+2.17}{4}\left(4-m\right)+\frac{{\text{MPG}}_{x}/35.6+2.17}{2}m\\ & =\frac{{\text{Hb}}_{1}}{4}\left(4-m\right)+\frac{{\text{Hb}}_{x}}{2}m=9.75\%. \end{array}$$

Furthermore, during the past one month, second group of RBCs have been newly formed and undergone glycation on the new curve (MPG = 137) with mean HbA_1c_ of

(11) $$\begin{array}{c} \Phi_{2}=\frac{{\text{MPG}}_{x}/35.6+2.17}{4}m=\frac{{\text{Hb}}_{x}}{4}m=1.5\text{\%}. \end{array}$$

Following all above steps, we can find the final desired HbA_1c_ by averaging equations Φ_1_ and Φ_2_ considering their coefficients according to available RBCs in each group (3 : 1, three months versus one month).

(12) $$\begin{array}{cc} {\text{Hb}}_{\text{mix}} & =\frac{\Phi_{2}\times m+\Phi_{1}\times \left(4-m\right)}{4},\\ {\text{Hb}}_{\text{mix}} & =\frac{\left(\left({\text{MPG}}_{x}/35.6+2.17\right)/4\right)m^{2}}{4}\\ & \;+\frac{\left(\frac{\frac{{\text{MPG}}_{1}}{35.6}+2.17}{4}\left(4-m\right)+\frac{\frac{{\text{MPG}}_{x}}{35.6}+2.17}{2}m\right)}{4}\\ & \;\times \frac{\left(4-m\right)}{4},\\ {\text{Hb}}_{\text{mix}} & =\frac{\left({\text{Hb}}_{x}/4\right)m^{2}+\left(\left({\text{Hb}}_{1}/4\right)\left(4-m\right)+\left({\text{Hb}}_{x}/2\right)m\right)}{4}\\ & \;\times \frac{\left(4-m\right)}{4}. \end{array}$$

And rearranging gives

(13) $$\begin{array}{c} {\text{Hb}}_{\text{mix}}=\frac{{\text{MPG}}_{x}\left(8m-m^{2}\right)+{\text{MPG}}_{1}\left(m^{2}-8m+16\right)+1236}{570}. \end{array}$$

or

(14) $$\begin{array}{c} {\text{Hb}}_{\text{mix}}=\frac{{\text{Hb}}_{x}\left(8m-m^{2}\right)+{\text{Hb}}_{1}\left(m^{2}-8m+16\right)}{16}=7.6875\text{\%}. \end{array}$$

Therefore, the estimated HbA_1c_ after one month would be 7.6875%. Although this is the answer to our initial riddle, this value (Hb_mix_) is a simply measurable variable by laboratory assays. In fact, our unknown desirable variable in this setting would be MPG*_x_* and Hb*_x_* representative of the second curve in which glycation occurs over recent months. And this is the finding that was one of the most important limitations of HbA_1c_, some minutes ago.

Access to the equations governing HbA_1c_ by this comprehensive analysis could have potentially valuable implications on diabetes control. No matter how frequently done, measurement of HbA_1c_ can lead to the desirable mean plasma glucose over previous *m* months and makes all doubts about time wasting over patient's observations, even. Finally, for practical aspects of this model in clinical setting,

(15) $$\begin{array}{c} {\text{MPG}}_{x}=\frac{570\times {\text{Hb}}_{\text{mix}}-{\text{MPG}}_{1}\times \left(m^{2}-8m+16\right)-1236}{\left(8m-m^{2}\right)}. \end{array}$$

or

(16) $$\begin{array}{c} {\text{Hb}}_{x}=\frac{16\times {\text{Hb}}_{\text{mix}}-{\text{Hb}}_{1}\times \left(m^{2}-8m+16\right)}{\left(8m-m^{2}\right)}, \end{array}$$

where

- Hb_1_: initially measured HbA_1c_,
- Hb_mix_: measured HbA_1c_ after *m* months,
- Hb_*x*_: HbA_1c_ corresponding to the curve on which the patient has moved during previous *m* months,
- *m*: time interval between measured Hb_1_ and Hb_mix_ in month.

It is of note that, the final equation (16) is independent of presumed equations correlating HbA_1c_ with MPG such as DCCT data ((1) and (2)) or other data such as Nathan's et al. [5]. The calculated Hb_*x*_ has a capability to be converted to the corresponding MPG using any of mentioned HbA_1c_-MPG relationships (Table 1).

**Example 2**

Assume a diabetic patient with Hb_1_ = 12% and MPG_1_ = 350 to whom changing in therapeutic regimens is applied. After two weeks, the rechecked HbA_1c_ is Hb_mix_ = 11%. According to (16), the mean plasma glucose in recent two weeks can be calculated as

(17) $$\begin{array}{c} {\text{Hb}}_{x}=\frac{16\times 11-12\times \left(0.25-4+16\right)}{\left(4-0.25\right)}=6.93\%. \end{array}$$

Hb_*x*_ = 6.93% represents that the patient is shifted to and moving on the curve MPG = 170 (see (1)) showing a significant improvement in patient's diabetic control. Otherwise, the measured Hb_mix_ = 11% corresponds to the MPG = 315 with a remarkable error and deviation from reality due to a mixture of former and later glycated Hemoglobins.

It is of note that, variation in glycation rates between individuals and also difference in RBC life span especially in hemoglobinopathies are not factored in this model to attenuate intricacy of equations.

**Figure 4:**
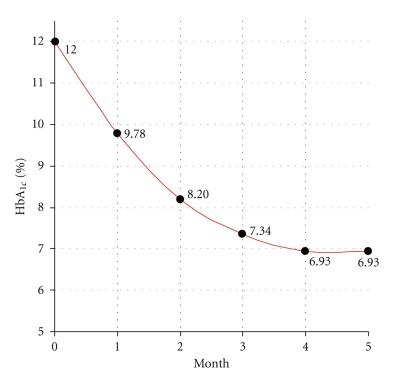
Changes in HbA_1c_ versus time for the patient presented in Example 2.

Another application of this derived mathematical model is describing the changes in HbA_1c_ with time. For the patient presented in Example 2, (14) takes the form of

(18) $$\begin{array}{c} {\text{Hb}}_{\text{mix}}=\frac{6.93\times \left(8m-m^{2}\right)+12\times \left(m^{2}-8m+16\right)}{16}. \end{array}$$

and can be plotted as in Figure 4.

Percentage of changes in HbA_1c_ during the time intervals can be expressed as

(19) $$\begin{array}{c} \Delta_{{\text{HbA}}_{1\text{c}}}=\frac{{\text{Hb}}_{1}-{\text{Hb}}_{\text{mix}}\left(\text{time}-\text{related}\right)}{{\text{Hb}}_{1}-{\text{Hb}}_{x}}\times 100. \end{array}$$

and is presented in Table 2.

The calculated changes of HbA_1c_ over time derived from devised mathematical model are in full quantitative agreement with previous clinical studies [11–13] showing that plasma glucose levels in the preceding 30 days contribute ~50% to the final results, and PG levels from 90–120 days earlier contribute only ~10%.

As briefly described, without applying the presented equations, early measurement of HbA_1c_ will end to a crude and erroneous estimation of patient's MPG. How frequently should it be checked is a great controversy among authorities, but the general trend and recommendation vary from 2 to 3 months.

Additional application of our mathematical model is calculation of emerged error at any desired time intervals, defined as deviation of the crude estimation of MPG derived via Hb_mix_, from real MPG calculated by devised equations

(20) $$\begin{array}{c} \text{error}=\frac{{\text{MPG}}_{\text{real}}-{\text{MPG}}_{\text{crude}}}{{\text{MPG}}_{\text{real}}}\times 100. \end{array}$$

or

(21) $$\begin{array}{cc} \text{error} & =1-\frac{\left(35.6\times {\text{Hb}}_{\text{mix}}-77.3\right)}{\left(\frac{570\times {\text{Hb}}_{\text{mix}}-{\text{MPG}}_{1}\times \left(m^{2}-8m+16\right)-1236}{\left(8m-m^{2}\right)}\right)}\times 100. \end{array}$$

To make the presented 3-variable equation more applicable, it can be used at definite points of time with different values of MPG_1_ and laboratory measured Hb_mix_. As an instance, error estimation of measured HbA_1c_ for detection of patient's mean plasma glucose in 2 and 3 months intervals is expressed in Tables 3 and 4 according to different values of MPG_1_ and Hb_mix_.

As presented in Tables 3 and 4, estimated error emerged in different values of MPG_1_ and Hb_mix_ ranges from −50% to +20% for 2-month interval and −26% to +5% for 3-month interval. Negative and positive errors contribute to overestimation and underestimation of patient's MPG, respectively. The higher the difference between Hb_1_ and Hb_mix_ is, the bigger the error emerged from crude estimation of patient's MPG via laboratory measured Hb_mix_.

## 3. Discussion

Hemoglobin is continuously glycated during the 120-day life span of erythrocyte such that the cumulative amount of HbA_1c_ in an erythrocyte is directly proportional to the time-averaged concentration of glucose within the erythrocyte [8, 10, 14, 15]. Glycated hemoglobins provide an index of the patient's average blood glucose concentration over a long time period. This index is not affected by short-term fluctuations in blood sugar (hour to hour) and hence gives a relatively precise reflection of the state of blood glucose control in diabetes.

To introduce novel applications and new concepts about HbA_1c_, an innovative mathematical simulation was analytically modeled to describe the HbA_1c_ behavior and process of events. The basic suppositions are cited from available equations expressed in Figures 1 and 2 [9, 10]. The devised model is used to predict the mean plasma glucose at any desired point in time with great certainty. By using derived formulas, it does not take 120 days to detect a clinically meaningful and reliable value for HbA_1c_ and MPG over preceding months.

In addition to the presented application, I specifically was interested in assessing the pattern of HbA_1c_ changes over time and calculation of emerged error during crude estimation of MPG from Hb_mix_. As described in Table 2 and Figure 4, change in HbA_1c_ shows a prompt fall upon institution of rigorous diabetic control. This finding can be readily explained by decay of older erythrocytes with highest rates of glycated Hb, as demonstrated in Figure 3. This model refutes the explanation that recent PG levels (i.e., 3-4 weeks earlier) contribute considerably more to the level of HbA_1c_ than do long past PG levels (i.e., 3-4 months earlier) [11–13].

According to calculated error for 2- and 3-month interval and its explained logic, without employment of the devised model, HbA_1c_ should be used with caution as a surrogate measure of MPG because it may significantly under or overestimate patient's MPG.

The tests currently in use for diagnosis are the fasting plasma glucose test and the less common oral glucose tolerance test. However, these tests can be inaccurate if a person has eaten recently or is sick. Advantages of the HbA_1c_ test are that it can be given at any time and, because it reflects blood glucose levels over a longer period, it is not unduly influenced by events on the day of the test. This devised model also makes HbA_1c_ more befitting and useful for being a main part of guidelines on using the HbA_1c_ test as a diagnostic tool for diabetes. However, a consensus statement is necessary because right now there is no agreement on what HbA_1c_ level would constitute a diagnosis of diabetes.

## Figures and Tables

**Table 1:** Regression-estimated MPG at different HbA_1c_ levels based on DCCT and Nathan's data.

HbA_1c_ (%)	DCCT-estimated MPG		ADAG-stimated MPG	
	mmol/l	mg/dl	mmol/l	mg/dl
5	5.6	101	5.4	97
6	7.6	137	7.0	126
7	9.6	172	8.6	154
8	11.5	208	10.2	183
9	13.5	244	11.8	212
10	15.5	279	13.4	240
11	17.5	315	14.9	269
12	19.5	350	16.5	298

**Table 2:** Percentage of changes in HbA_1c_ during time intervals.

Month	First	Second	Third	Forth
During	44%	31%	17%	8%
Total changes at the end of	44%	75%	92%	100%

**Table 3:** MPG_1_ versus Hb_mix_ showing estimated error of measured HbA_1c_ for detection of patient's MPG in 2-month intervals. Out of range data are ignored.

Error2	101	137	172	208	244	279	315	350	385	420
5	0	−12.8	−29.5	−52.9						
6	8.32	0	−8.83	−20.2	−34.3	−51.6				
7	12.37	6.723	0	−6.86	−15.4	−25.1	−36.9	−50.8		
8	14.84	10.48	5.785	0	−5.61	−12.2	−19.9	−28.5	−38.4	−49.9
9	16.51	12.96	9.203	4.987	0	−4.59	−10.2	−16.3	−23.1	−30.8
10	17.71	14.72	11.59	8.129	4.385	0	−3.97	−8.65	−13.8	−19.4
11	18.61	16.03	13.35	10.42	7.281	4.01	0	−3.39	−7.47	−11.9
12	19.32	17.04	14.71	12.16	9.465	6.676	3.622	0	−2.93	−6.55
13	19.88	17.85	15.78	13.54	11.17	8.742	6.102	3.383	0	−2.55
14	20.35	18.52	16.65	14.65	12.54	10.39	8.067	5.689	3.184	0

**Table 4:** MPG_1_ versus Hb_mix_ showing estimated error of measured HbA_1c_ for detection of patient's MPG in 3-month intervals.

Error3	101	137	172	208	244	279	315	350	385	420
5	0	−2.27	−4.76	−7.44	−10.3	−13.2	−16.3	−19.5	−22.9	−26.5
6	1.846	0	−1.62	−3.47	−5.39	−7.33	−9.4	−11.5	−13.7	−15.9
7	2.806	1.469	0	−1.28	−2.73	−4.19	−5.73	−7.27	−8.85	−10.5
8	3.427	2.336	1.25	0	−1.06	−2.22	−3.45	−4.66	−5.91	−7.19
9	3.861	2.939	2.026	1.069	0	−0.88	−1.89	−2.9	−3.92	−4.97
10	4.182	3.384	2.596	1.772	0.933	0	−0.76	−1.62	−2.49	−3.38
11	4.428	3.725	3.032	2.308	1.574	0.849	0	−0.65	−1.41	−2.18
12	4.624	3.996	3.377	2.732	2.078	1.434	0.763	0	−0.57	−1.25
13	4.783	4.215	3.656	3.074	2.485	1.906	1.303	0.71	0	−0.5
14	4.914	4.396	3.886	3.357	2.821	2.295	1.747	1.209	0.665	0
